# Supplementary material for: The computation of directional selectivity in the Drosophila OFF motion pathway
Source: eLife. 2019 Dec 11;8:e50706. doi: 10.7554/eLife.50706 (PMC6917495; doi:10.7554/eLife.50706)
Supplement: Supplementary file 1. [file elife-50706-supp1.docx]

| Parameter | Description | Units | Bounds |
| --- | --- | --- | --- |
| 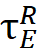 | Rise time of E conductance | ms | 1 - 400 |
| 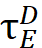 | Decay time of E conductance | ms | 1 – 400 |
| 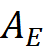 | Conductance amplitude (E) | unitless | 0 – 10 |
| 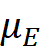 | Receptive field location (E) | unitless | -5 – 5 |
| 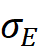 | Width of conductance spatial profile (E) | unitless | 0 – 10 |
| 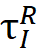 | Rise time of I conductance | ms | 1 - 400 |
| 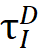 | Decay time of I conductance | ms | 1 - 400 |
| 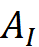 | Conductance amplitude (I) | unitless | 0 – 10 |
| 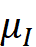 | Receptive field location (I) | unitless | -5 – 10 |
| 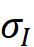 | Width of conductance spatial profile (I) | unitless | 0 - 10 |
| V_E_ | Reversal potential (E) | mV | 0 (fixed) |
| V_I_ | Reversal potential (I) | mV | -74 (fixed) |
| V_L_ | Resting potential | mV | -65 (fixed) |

**Excitation-Inhibition model parameters (related to Figures 4, 5, 7).**
