# Supplementary material for: The computation of directional selectivity in the Drosophila OFF motion pathway
Source: eLife. 2019 Dec 11;8:e50706. doi: 10.7554/eLife.50706 (PMC6917495; doi:10.7554/eLife.50706)
Supplement: Supplementary file 2. [file elife-50706-supp2.docx]

| Parameter | Description | Units | Bounds |
| --- | --- | --- | --- |
| 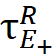 | Rise time of conductance 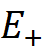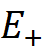 | ms | 1 - 400 |
| 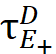 | Decay time of conductance 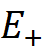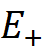 | ms | 1 – 400 |
| 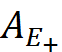 | Conductance amplitude () 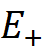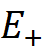 | unitless | 0 – 10 |
| 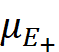 | Receptive field location () 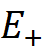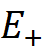 | unitless | -5 – 5 |
| 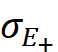 | Width of conductance spatial profile () 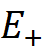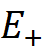 | unitless | 0 – 10 |
| 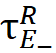 | Rise time of conductance 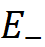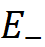 | ms | 1 - 400 |
| 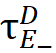 | Decay time of conductance 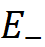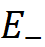 | ms | 1 - 400 |
| 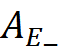 | Conductance amplitude () 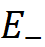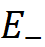 | unitless | 0 – 20 |
| 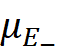 | Receptive field location () 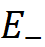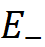 | unitless | -5 – 10 |
| 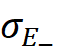 | Width of conductance spatial profile () 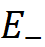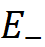 | unitless | 0 - 10 |
| 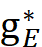 | stimulus-independent E conductance | unitless | 0 - 20 |
| 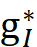 | stimulus-independent I conductance | unitless | 0 - 100 |
| V_E_ | Reversal potential (E) | mV | 0 (fixed) |
| V_I_ | Reversal potential (I) | mV | -74(fixed) |
| V_L_ | Resting potential | mV | -65 (fixed) |

**Excitation-Removal of excitation model parameters (related to Figures 6, 7).**
